# Evaluation of Oogenesis Aspects in Neonatal and Adult Mice after Toloaldoxime Treatment

**DOI:** 10.22074/cellj.2015.18

**Published:** 2015-10-07

**Authors:** Mohammad Fazeltabar Malekshah, Mahsa Sedighi, Kazem Parivar, Homa Mohseni Kouchesfahani, Mohamadali Bigdeli

**Affiliations:** 1 Department of Embryology, Reproductive Biomedicine Research Center, Royan Institute for Reproductive Biomedicine, ACECR, Tehran, Iran; 2 Faculty of New Science and Technology, Tehran University, Tehran, Iran; 3 Department of Biology, Faculty of Science, Science and Research Branch, Islamic Azad University, Tehran, Iran; 4 Faculty of Chemistry, Tarbiat Moalem University, Tehran, Iran

**Keywords:** Toloaldoxime, Ovary, Balb/c Mouse, Fertility

## Abstract

**Objective:**

Oximes are important materials in organic chemistry. Synparamethyl benzal- dehyde oxime (toloaldoxime) is structurally similar to other oximes, hence we have studied its effects on the neonatal and adult female Balb/c mice reproductive systems in order to provide a platform for future studies on the production of female contraceptive drugs.

**Materials and Methods:**

In experimental study, we studied the effects of toloaldoxime on ovary growth and gonadal hormones of neonatal and adult Balb/c mice. A regression model for prediction was presented.

**Results:**

The effects of toloaldoxime on neonatal mice were more than adult mice. The greatest effect was on the number of Graafian follicles (59.6% in adult mice and 31.83% in neonatal mice). The least effect was on ovary weight, and blood serum lev- els of follicle stimulating hormone (FSH) and luteinizing hormone (LH).

**Conclusion:**

According to the data obtained, toloaldoxime can be considered an anti- pregnancy substance.

## Introduction

Currently, an optimal population with high efficiency is required for economic growth. In society, the level of education and specialty determines the level of efficacy. Unfortunately, in most developing countries the social and economic development programs remain behind schedule due to overwhelming population growth. Therefore, population growth control is a vital necessity. Fortunately, the misconception that only women are responsible for contraception is gradually changing and studies are focusing on male contraceptives. However, the main focus is still on women as targets for contraception. Anti-fertility effects of a number of drug groups such as Kraven Ether on female fertility have been studied (1). 

Oximes are a group of organic chemicals produced in high volumes with industrial applications. These chemicals are conventionally divided in two groups ketoximes and aldoximes (2,3). Oximes are converted to oxime salts in both acidic and alkali environments. Therefore, they are soluble in both media (4). Characteristics of these chemicals include their effects on human behavior (4), industrial implications (5), anti-cancer effects (1,6,7), as well as anti-convulsion, anti-microbial, and anti-inflammatory characteristics, and involvement in enzymatic reactions (8). 

In 1996, researchers observed a significant difference between test group mice (B6c3f) that received cyclohexaton oxime and the control group in terms of weight, estrous cycle days and stages. There was also a significant difference between the two groups based on the number of spermatids and sperm (8,9). The anti-fertility characteristics of these chemicals have also been tested in men and women. Norethisterone-3-oxime-acetate is an oxime chemical with clinical applications as a contraceptive pill. Studies have shown that this chemical is an active contraceptive tablet with numerous benefits, very few side effects (9,11). 

On the other hand, tiforoxime is an oxime from the tiroforans family that has anti-protozoa and antimicrobial effects. This substance has been tested in adult rats. The results showed a significant decrease in sperm motility, epididiomal sperm storage, and fertility rate in the test group compared to the control group. Testosterone production was unaffected (12). 

Different parts of the female reproductive system have different sensitivity levels to various substances and drugs; some drugs assert their effects in specific parts of this system. Therefore, different criteria must be considered to study the effects of a substance on the reproductive system in order to determine which part of the system is affected by this substance. 

In this study, we evaluated the anti-fertility effects of toloaldoxime, a member of the oxime family, as a potential future contraceptive for women. The effects of toloaldoxime on ovarian growth in both immature and mature mice was studied and assessed according to the following 24 criteria: increase in body weight, ovary weight, relative ovary weight; macroscopic and microscopic ovarian diameters; the numbers of yellow bodies, their diameters, and the numbers of cells in the yellow bodies; the numbers of primordial follicles, primary follicles, intact Graafian follicles, growing follicles, and atrophic Graafian follicles; the diameters of primary oocytes; atrophic Graafian follicles; the thickness of intact granulosa layers and atrophic Graafian follicles; the thickness of the theca layer in intact and atrophic Graafian follicles; and the concentrations of follicular stimulating hormone (FSH), luteinizing hormone (LH), estrogen, and progesterone in blood serum. 

## Materials and Methods

### Preparation of toloaldoxime

Toloaldoxime (98%) was provided by the Chemistry Faculty at Kharazmi University under an experimental study. The determined lethal dose 50 (LD_50_) in these experiments was 325 mg/kg body weight (BW). Adult mice received 140 mg/kg BW and immature mice received 110 mg/kg BW which equaled 0.14 mg/g mouse weight or 0.0056 cc. This project was received approval from the Ethical Committee of Kharazmi University, Tehran, Iran. 

### Animals

We used Balb/c mice purchased from Pasteur Institute, Iran. The mice were kept in special cages at the animal house of Royan Institute. Food for the mice was purchased from Pars Company for birds and animals. Temperature and humidity of the animal house was adjusted. In order to establish dark and light cycles, we used an electrical timer at 12 hours. Under these conditions the mice were coupled by the polygamy method; each male was placed in a cage with 2 to 3 adult female mice. After 30 days of infancy, the offspring were separated from their mothers and raised separately (11). 

### Short-term injection

For this method, we used mature adult virgin female mice (10-12 weeks). The injection was performed daily over a 10-day period. At 48 hours after the last injection, the mice were anesthetized and their blood was collected. Their ovaries were removed, measured, and used for histological analyses. Mice were divided into the following groups: i. Test group received intraperitoneal injections of toloaldoxime based on their body weights, ii. Sham group received intraperitoneal injections of olive oil and iii. Control group, which were raised naturally compared to the test and sham groups. 

### Long-term injection

Immature mice (4-5 weeks) were injected every other day over a 20-day period. At 10 days after the last injection, after maturation, the mice were anesthetized. Their blood was collected and their ovaries were removed, weighed and measured prior to histological analyses. The groups were as follows: i. test group received intraperitoneal injections of toloaldoxime based on their body weight, ii. sham group received intraperitoneal injections of olive oil and iii. control group were raised naturally compared to the test and sham groups. This group was the basis for comparison between groups. 

### Histological analyses

The ovaries were sectioned, then stained with Toloaldoxim Ef fects on Mice Oogenesis hematoxylin and eosin. Stained sections were studied with a light microscopy (12). All ovaries in the test groups were compared with each other. All statistical measurements were performed with SPSS version 13 software. 

## Results

**Fig.1:**
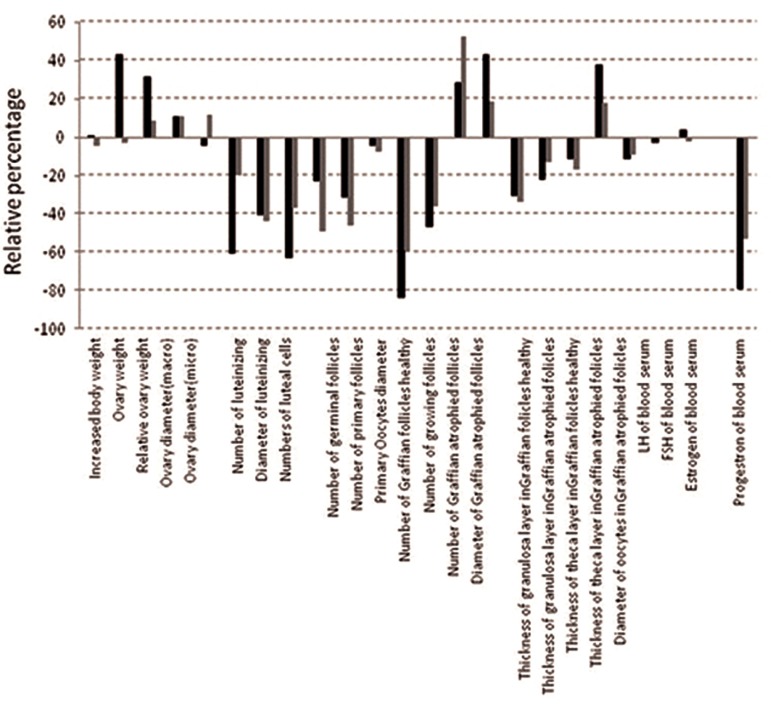
Comparison of relative experimental changes and controls in mature and immature mice according to 24 different parameters. FSH; Follicle stimulating hormone and LH; Luteinizing hormone.

We investigated the effects of toloaldoxime on mice. Figure 1 shows the difference between the test and control groups in both mature and immature Balb/c mice. We observed a significant increase based on 6 criteria (the positive amount of relative difference), a significant decrease in 14 parameters (the negative amount of relative difference) and no difference in 4 parameters (results ± 4%) in the test group compared to the control group. In terms of relative difference between the control and sham groups, there was a significant increase in 6 parameters (the positive amount of relative changes), a significant decrease in 11 parameters (the negative amount of relative changes) and no significant difference in 7 parameters (results ± 4%). Toloaldoxime had the most effect on the numbers of Graafian follicles (59.64% in mature mice and 31.81% in immature mice) and the least effect on ovarian weight, and serum levels of LH and FSH.

**Fig.2:**
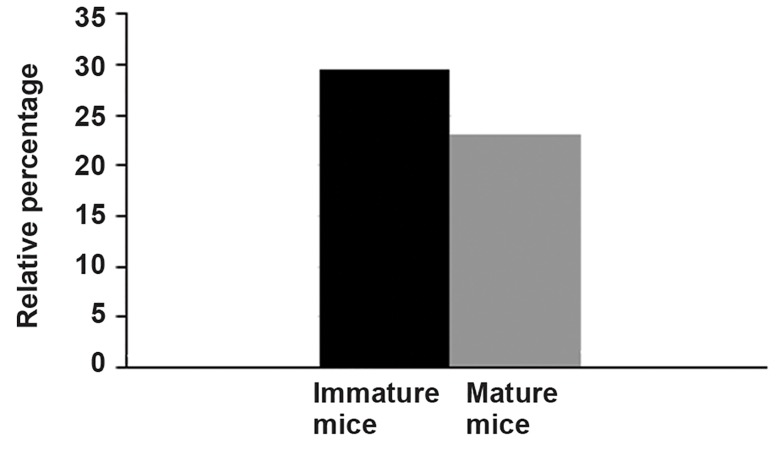
Relative percent changes of total experimental parameters compared to controls for mature and immature mice.

The experimental effect on all parameters showed that the relative percent change in all parameters in the test group compared to the control group in immature mice (29.52%) was approximately 1.2 times that of mature mice (12.23%). Figure 2 shows that the experimental changes in immature mice are more than seen in mature mice.

**Fig.3:**
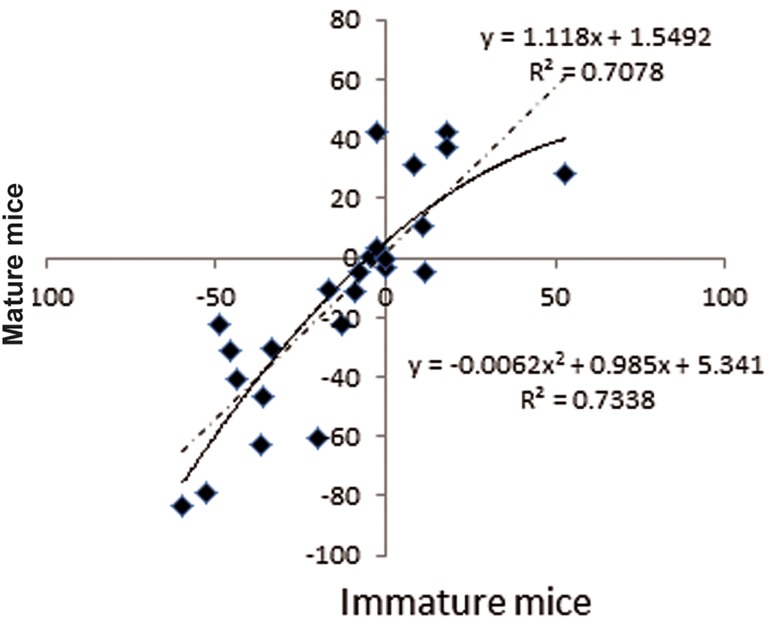
Regresion model fit of relative percent changes of experimental and sham parameters. y; Immature mice, x; Mature mice and R^2^; Determination fraction.

The relation between the relative percent of two variants in experimental parameters and sham parameters are shown in figure 3. The relative percent change in experimental parameters to control parameters in mature mice is half of the percent change seen in immature mice.

## Discussion

Due to the similar structure of toloaldoxime with other oximes, this study was designed to evaluate the effects of toloaldoxime on oogenesis in order to ascertain a possible female contraceptive compound with minimum side effects. The LD_50_ for toloaldoxime was determined to be 325 mg/kg BW. In this research, mature and immature mice received a single intraperitoneal dose of toloaldoxime as 140 mg/kg BW for 10 consecutive days or 110 mg/kg BW for 20 alternate days. On the 12^th^ day after the first injection in mature mice and the 30th day after the first injection in immature mice, the animals were killed and their ovarian sections studied under a light microscope.

The results showed that in the mature experimental group there were significant decreases compared with controls in the parameters of body weight; numbers of primordial, primary, growing and intact Graafian follicles; granulosa layer thickness; and blood level of progesterone. We observed a significant increase compared with the control group in parameters of thickness of the theca layer in atretic Graafian follicles, ovary diameter, and the number of atretic Graafian follicles. However there were no significant changes in parameters of ovarian weight and its ratio to body weight and number of corpus luteum, corpus luteal cells and diameter of corpus luteum and primary oocytes, oocytes in atretic Graafian follicles and thickness of granulosa cell layer in atretic Graafian follicles, theca layer in intact Graafian follicles and blood levels of LH and FSH. Serum estrogen levels were stable in all groups.

In immature mice there was significant decrease in the parameters of body weight; number of growing follicles; intact Graafian follicles; thickness of granulosa layer in intact Graafian follicles; and blood level of progesterone hormone compared with the control group. A significant increase was observed in the parameters of ovary weight, thickness of the theca layer of atretic Graafian follicles, and diameter of atretic Graafian follicles compared with controls. However the parameters of relative ovary weight, number of corpus luteum; corpus luteal cells; primordial follicles; atretic Graafian follicles; primary follicles; and diameters of ovary, corpus luteum, primary oocyte and atretic Graafian follicle oocyte; as well as the thickness of the granulosa layer in atretic Graafian follicles, theca layer in intact Graafian follicles; and blood level of LH and FSH did not significantly change. Estrogen blood levels were stable in all groups.

The relative percent of experimental and sham parameters were evaluated using one variant regression and value engineering methods. In numerous problems, there are two or more parameters internally related to each other where the identity of this relation has to be clear. Regression analysis is a statistical technique for modeling and studying the relation between two or more variants. The amount of determination fraction (R^2^) is a good criterion for evaluating the model and the closer this value is to 1, the better the model (12).

As shown in figure 3, the evaluated multisentence model has an R^2^ value of 0.73 which shows its authenticity. The foretelling model, the power of the relative changes in experimental parameters to the control group in immature mice (y) from the relative changes in experimental changes to the control group in mature mice (x) is shown as y=-0.0062x2+0.985x+5.341. The linear regression model for determining the line slope among the estimated data is shown as y=1.118x+1.5492. The amount of the line slope is 1/118 which shows that the amount of the relative change percent in experimental parameters to control parameters in mature mice is generally ½ of that of immature mice.

## Conclusion

Based on the data obtained from studying the effect of toloaldoxime on oogenesis and gonadal hormones in both immature and mature Balb/c mice, we can consider toloaldoxime to be an anti-tumor and an anti-pregnancy substance. The effect of toloaldoxime on oogenesis and gonadal hormones in immature mice is three times more than its effects on that of mature mice. We have observed the highest effect of toloaldoxime on the number of intact Graafian follicles whereas the lowest effect was on ovarian weight, and the levels of LH and FSH in blood serum. Current work of that uses toloaldoxime for preparing cancer therapeutics is limited to "proof-of-concept" studies. Extended research is necessary before this organic chemical can be of use in clinical practice.
